# Maxillary canine‒second molar transposition: A rare case report

**DOI:** 10.15171/joddd.2017.024

**Published:** 2017-06-21

**Authors:** Somayeh Hekmatfar, Karim Jafari, Firoz Zadfatah, Sahar Mousavi

**Affiliations:** ^1^Department of Pediatric Dentistry, Dental Faculty, Ardabil University of Medical Sciences, Ardabil, Iran; ^2^Department of Prosthodontics, Dental Faculty, Ardabil University of Medical Sciences, Ardabil, Iran; ^3^Department of Endodontics, Dental Faculty, Ardabil University of Medical Sciences, Ardabil, Iran

**Keywords:** Maxillary canine, transposition, thalassemia

## Abstract

Tooth transposition, which is a rare condition, is ascribed to the disturbance of tooth eruption and the subsequent abnormal occlusal relationships. Transpositions mostly involve the upper jaw and more frequently occur between the maxillary canine and first premolar. Herein, we present a case of a maxillary canine‒second molar transposition in a thalassemic patient.

## Introduction

Tooth transposition is a rare condition, which refers to the disturbance of tooth eruption and the subsequent abnormal occlusal relationships.^1^The incidence rate of this condition differs based on the sample studied; however, in the majority of the case studies, this rate has been reported to be under 1%.^2-4^ Transpositions mostly involve the upper jaw and are more frequently seen between the maxillary canine and first premolar.^5-6^Canine transposition mostly occurs in the females.^6-7^

Peck and Peck categorized the maxillary transposition into five types, arranged by incidence, including canine‒first premolar, canine‒lateral incisor, canine‒first molar, lateral incisor‒central incisor, and canine‒central incisor.^6^ The canine transposition is a multifactorial condition, which is associated with both genetic and environmental factors.^8^ Several risk factors have been identified for this dental anomaly, including the positional interchange of tooth buds,^7-9^deviation from the normal eruption path,^10^existing primary teeth,^11^ and trauma.^12^ In addition, some medical conditions and systemic diseases, such as blood dyscrasia, may lead to this condition.

Fanconi anemia is a rare genetic disease caused by decreased blood cells, which affects the bone marrow. This disease is associated with dental anomalies. According to Goswami et al, microdontia, supernumerary teeth, tooth agenesis, discolored teeth, abnormal tooth shape, rotation, transposition and delayed eruption are among the most common dental anomalies observed in patients with Fanconi anemia.^13^ Tekcicek et al^24^ reported that transposition occurred in 9% of the children with Fanconi anemia.

Thalassemia is a hereditary chronic microcytic anemia described by deficiency in hemoglobin synthesis and ineffective** **erythropoiesis. This disease is one of the most confusing hemoglobinopathies.^14^ According to Khojastehpour et al, dental anomalies are more common in thalassemic patients than in normal individuals. The aim of this paper is to report a case of a maxillary canine‒second molar transposition in a thalassemic patient.

## Case report

A 22-year-old woman referred to the Ardabil Dental Faculty Clinic, Ardebil, Iran, with a chief complaint of pain in the upper first molar area. The patients’ medical history revealed that she was a known case of β-thalassemia minor. She had undergone splenectomy five years ago without any history of blood transfusion. Due to high platelet counts, aspirin was recommended by an oncologist to reduce the risk of thromboembolism.

**Figure 1:**
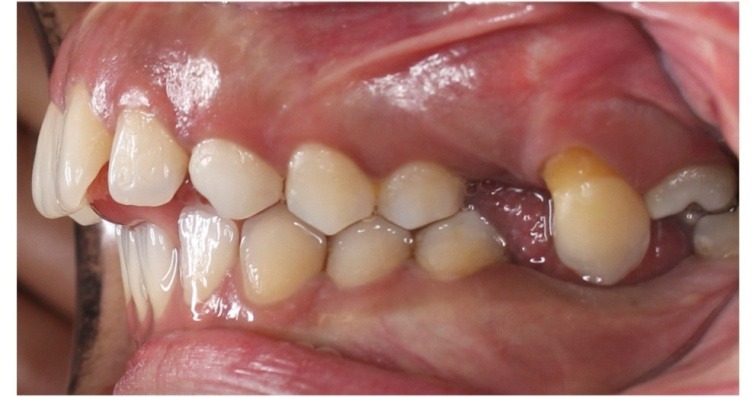
Intraoral view of the canine erupting mesial to the third molar.

**Figure 2:**
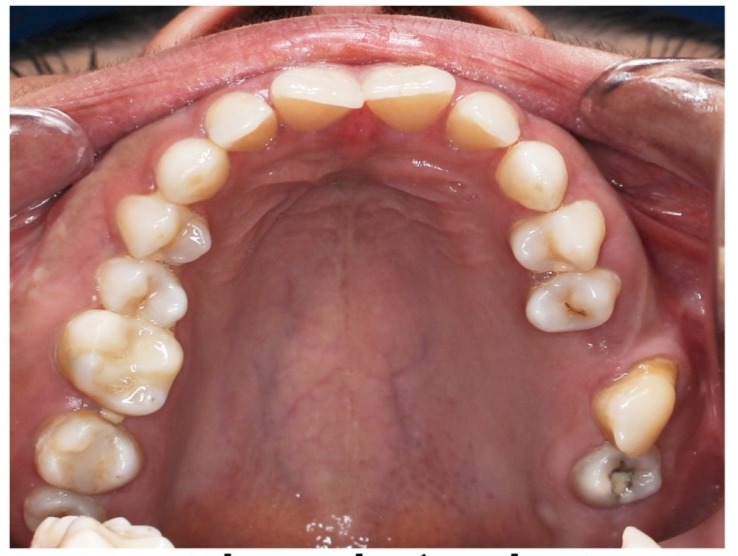
Occlusal view of the transposed maxillary canine.

The intraoral examination revealed the transposition of the left maxillary canine that erupted mesial to the third molar (Figure 1). The distal slope of the canine was in contact with the mesial marginal ridge of the lower left third molar. The crown of the transpositioned canine was well-formed with a prominent cingulum (Figure 2).

**Figure 3:**
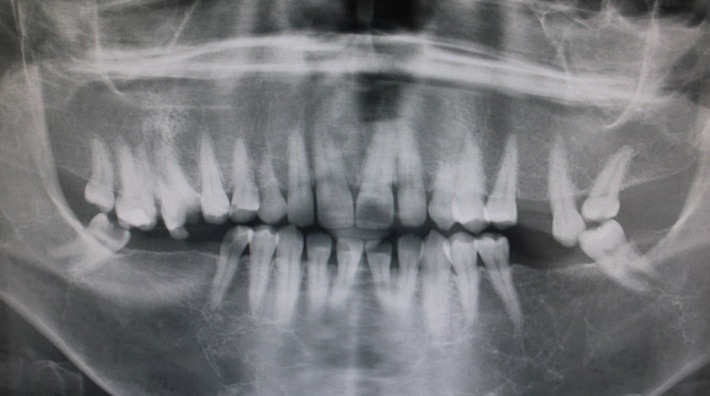
Panoramic view of the enlargement of marrow spaces with widened trabeculae.

The left maxillary first and second molars as well as the right and left mandibular first and second molars were missing. Furthermore, major occlusal plane discrepancies were observed due to the mandibular posterior teeth lost. The right canine was also missing. The patient interview revealed that the mentioned tooth was impacted and already surgically extracted by an oral and maxillofacial surgeon. The upper and lower dental midlines did not coincide, and there were spaces between the lower anterior teeth.

The right and left deciduous maxillary canines were retained. The rare occasional finding in this patient was the transposition of the left canine that erupted mesial to the third molar (Figure 1). The left first and second molars were absent. The patient did not remember anything about these teeth.

**Figure 4:**
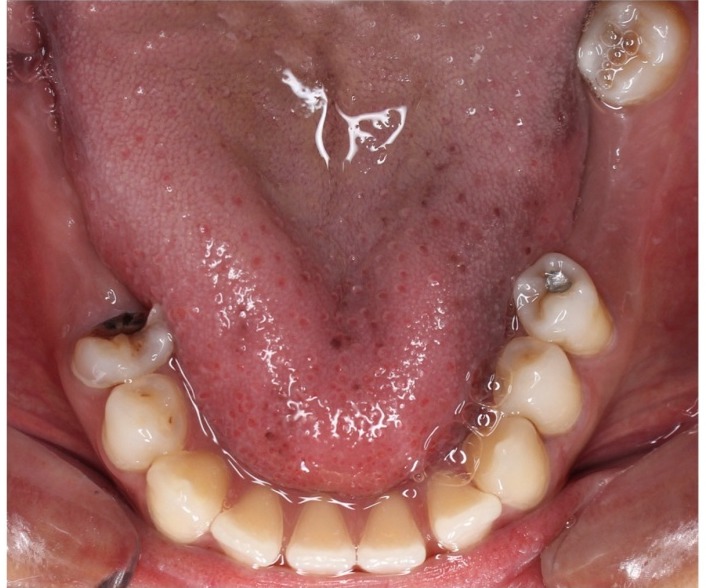
Macroglossia in a thalassemic patient.

The patient underwent radiological examination. The panoramic views revealed the enlargement of marrow spaces with widened trabeculae in both the maxilla and mandible (Figure 2). The soft tissue drapes had normal color and contours. The only abnormal finding in soft tissue examination was macroglossia (Figure 4).

To determine an appropriate treatment plan, an impression was made of both dental arches. The stone casts were mounted on a semi-adjustable articulator by the means of a facebow. The analysis of the mounted casts showed a severe occlusal plane discrepancy as well as over-erupted right second premolar and first molar that had contact with the lower edentulous ridge.

In this condition, the prosthodontic treatment modality necessitates administration of extensive orthodontic and surgical interventions to create adequate space for the replacement of the missing tooth. However, due to economic issues, the patient did not accept any prosthodontic treatment and only received endodontic** **therapy for the maxillary first molar.

## Discussion

Tooth transposition is a form of ectopic eruption, which can affect the maxillary or mandibular arch. This dental anomaly frequently involves the canine and first upper premolar.^5-6^,The unilateral dental transpositions are more prevalent than the bilateral ones and mostly affect the left side.^1,5^ No transposition has been reported in the deciduous dentition yet.^5,13^

Tooth transposition can be complete and incomplete based on the position of the roots and the crowns of transposed teeth. While the complete transposition denotes the involvement of both the crown and root in the transposed position, the incomplete type represents the transposition of either the crown or the root apices.^5^ According to Peck’s classification (1995), there are five types of tooth transposition, depending on the teeth involved.^6^

In our case, we observed a complete transposition of the left maxillary canine, which was reported to have a high incidence in previous studies. However, regarding the study by Peck, the canine‒second molar transposition has not been reported in the literature. In a study conducted by Shanmugasundaram, the canine was reported to erupt buccal to the contact area between the left second premolar and the first molar.^15^ Furthermore, Kayipmaz reported a case of a 21-year-old man with a left maxillary canine‒molar transposition.

β-thalassemia is due to the diversity of genetic defects resulting in diverse hematologic and clinical features such as anemia. There are several different genotypes that may present similar clinical manifestations. Accordingly, Margot reported such manifestations as generalized rarefaction of the alveolar bone, thinning of cortical bone, “chicken-wire” appearance of the widened bone marrow spaces, and large trabeculae in radiographic examinations.^23^ We also observed the enlargement of marrow spaces with widened trabeculae in both the maxilla and mandible. Other dental anomalies, which may occur along with tooth transposition, include agenesis or malformation of lateral incisors and the retention of deciduous teeth.^14^

In our case, the primary canines were retained on the left and right sides. The permanent canine of the left side erupted in transposed position, and the canine on the contralateral side was surgically extracted. The maxillary lateral incisors were normal in size and shape. Tooth transposition is a multifactorial condition, which occurs as a result of environmental and genetic factors with complex relationships.^8^

The administration of radiography is a critical measure in the early diagnosis of tooth transposition, which can greatly affect prevention of eruption disturbances. Depending on the position of the tooth, it can erupt in an ectopic position or remain impacted. In our case, an early intervention could prevent the impaction of the right maxillary canine.

Two therapeutic approaches have been proposed for the management of transposition, namely interceptive and definitive treatment. The decision-making on transposition treatment depends on the time of diagnosis of this condition.^6,15^The interceptive treatment can be carried out on the patients within 6‒8 years of age prior to the completion of transposition. The corrective measures for the eruptive path of the upper canines include extraction of retained deciduous teeth, positioning of the permanent lateral incisor in its physiological position, and maintenance of the space for the permanent canine.^15,16^

On the other hand, the definitive treatment consists of three phases, namely the extraction of one of the transposed teeth, alignment of the teeth in the transposed and correct position, as well as orthodontic correction.^17,18^

## Conclusion

The dental management of medically compromised patients, such as those with thalassemia, needs special attention. Therefore, it seems essential to employ a multidisciplinary approach involving a dental surgeon, a hematologist, an orthodontist, and a prosthodontist to safely provide these patients with seemingly benign blood dyscrasia with dental treatment. Nevertheless, many factors may affect the treatment outcomes, including esthetics, occlusion, dental crowding, treatment period, patient motivation and cooperation, as well as periodontal support of the transposed teeth.^1^

## Acknowledgments

The authors would like to thank the Vice Chancellor for Research Affairs of Dental Faculty of Ardabil University of Medical Sciences for their support.

## Authors’ contributions

KJ and SH performed the radiographic and clinical examinations as well as the literature review. SH drafted the manuscript and prepared the figures. FZ and SM critically revised the manuscript. All authors read and approved the final manuscript.

## Funding

The study was self funded.

## Competing interests

The authors declare no competing interests with regards to the authorship and/or publication of this article.

## Ethics approval

Patient reported in the study have given signed written consents for use of the photographs.
